# The distribution pattern and growth factor level in platelet-rich fibrin incorporated skin-derived mesenchymal stem cells: An *in vitro* study

**DOI:** 10.14202/vetworld.2020.2097-2103

**Published:** 2020-10-07

**Authors:** Igo Syaiful Ihsan, Deya Karsari, Nora Ertanti, Aristika Dinaryanti, Alexander Patera Nugraha, Purwati Purwati, Sri Agus Sudjarwo, Fedik Abdul Rantam

**Affiliations:** 1 Master Student of Vaccinology and Immunotherapeutica, Veterinary Medicine Faculty, Airlangga University, Surabaya, Indonesia; 2 Stem Cell Research and Development Center, Airlangga University, Surabaya, Indonesia; 3 Doctoral Student of Medical Science, Faculty of Medicine, Airlangga University, Surabaya, Indonesia; 4 Department of Health, Vocational Faculty, Airlangga University, Surabaya, Indonesia; 5 Department of Pharmacology, Veterinary Medicine Faculty, Airlangga University, Surabaya, Indonesia; 6 Department of Microbiology, Virology Laboratory, Veterinary Medicine Faculty, Airlangga University, Surabaya, Indonesia

**Keywords:** growth factor, platelet-rich fibrin, rabbit, skin mesenchymal stem cells

## Abstract

**Background and Aim:**

A skin wound in an animal must be cared for to prevent further health issues. Platelet-rich fibrin (PRF) and skin-derived mesenchymal stem cells (SMSCs) have been reported to have potential in increasing the rate of wound healing. This study aimed to analyze the distribution patterns and levels of platelet-derived growth factor (PDGF), insulin-like growth factor (IGF), vascular endothelial growth factor (VEGF), and transforming growth factor-β (TGF-β) in PRF incorporated with SMSCs.

**Materials and Methods:**

This study employed a true experiment (*in vitro*) design with post-test only performed in the control group alone. PRF and SMSCs were extracted from the blood and skin of 16 rabbits. SMSCs were characterized using immunocytochemistry to examine clusters of differentiation for 45, 73, 90, and 105. PRF was incorporated into the SMSCs and then divided into four groups (N=32/n=8): Group A (PRF only), Group B (PRF+SMSCs, incubated for 1 day), Group C (PRF+SMSCs, incubated for 3 days), and Group D (PRF+SMSCs, incubated for 5 days). Scanning electron microscopy was used to examine the distribution pattern of SMSCs between groups. The supernatant serum (Group A) and supernatant medium culture (Group D) were collected for the measurement of PDGF, IGF, VEGF, and TGF-β using an enzyme-linked immunosorbent assay sandwich kit. An unpaired t-test was conducted to analyze the differences between Groups A and D (p<0.01).

**Results:**

Group D had the most morphologically visible SMSCs attached to the PRF, with elongated and pseudopodia cells. There was a significant difference between the levels of growth factor in Groups A and D (p=0.0001; p<0.01).

**Conclusion:**

SMSCs were able to adhere to and distribute evenly on the surface of PRF after 5 days of incubation. The PRF incorporated SMSCs contained high levels of PDGF, IGF, VEGF, and TGF- β, which may prove to have potential in enhancing wound healing.

## Introduction

A skin wound is defined as a break in the continuity of skin, mucous membrane, or tissue surface caused by physical, chemical, or biological agents [1]. These injuries may result in further health issues and a poorer quality of life when left untreated [2]. When treating exotic animal species’ wounds, special consideration for the given animal’s behavior, unique anatomy, and tendency toward secondary stress-related health problems must be taken [3]. Two common complications of the skin wound healing process are fibrosis and chronic inflammation [4].

In addition, there are many wound care products that potentially increase the rate of wound healing. One of these products includes platelet-rich fibrin (PRF), which is a product of platelet concentration through blood centrifugation without anticoagulation [5]. During centrifugation, platelets are activated and trapped within the fibrin matrix, resulting in the production of a set of growth factors (GF). GF, some of which include the platelet-derived GF (PDGF), insulin-like GF (IGF), transforming GF (TGF), and vascular endothelial GF (VEGF), are essential components for various cellular functions [6,7]. PRF contains mitogenic and chemotactic compounds that promote and modulate cellular proliferation and attractants [8,9]. PRF can be polymerized, shaped into a three-dimensional solid structure, and used as a scaffold [10]**.** In addition, PRF can enhance the expression of various osteogenic differentiation markers, such as bone alkaline phosphatase, osteocalcin, osteopontin, and osteonectin in mesenchymal stem cells (MSCs) cultured in an osteogenic medium [11-14]. In regenerative medicine, MSCs have been postulated to have therapeutic effects [15]. Skin has been considered a potentially abundant source of multipotent adult stem cells, which have high levels of durability and rates of replication [16]. Skin-derived MSC (SMSCs) are thus a promising source of stem cells due to their high multipotency and proliferation characteristics, which are both useful in regenerative medicine and tissue engineering [17].

PRF and stem cell therapies will continue to gain prominence. A previous study [18] reports great potential of PRF and MSCs in the treatment of muscle injuries in a rabbit model because they were able to stimulate the proliferation and differentiation of cells. This has been attributed to the fact that PRF contains abundant GF and a three-dimensional structure that would be regarded as a suitable natural material for the seeding of SMSCs.

We hypothesized that SMSCs would attach to PRF, thus improving the GF levels further. The aim of this study was to analyze the distribution patterns and levels of PDGF, IGF, VEGF, and TGF-β in PRF incorporated with SMSCs. Furthermore, the prospective outcome of this study should better describe how PRF incorporated with SMSCs could be used as a treatment in veterinary regenerative medicine.

## Materials and Methods

### Ethical approval

This study was approved by the Research Ethics Committee on the Use of Animals, Faculty of Veterinary Medicine, Universitas Airlangga, with appointment number 289/HRECC.FODM/ XII/20170.

### Study period and location

This study was conducted from October 2019 to February 2020 at Stem Cell Research and Development Center, Airlangga University, Surabaya, Indonesia and Laboratory for Energy and the Environment, Institut Teknologi Sepuluh Nopember, Surabaya Indonesia.

### Study animals 

Sixteen 7-9-month-old, healthy, male New Zealand white rabbits (*Oryctolagus cuniculus*), each weighing 2.5-3.5 kg, were used as the isolation for this study PRF and SMSCs. Rabbits were acquired from the Stem Cell Research and Development Center, Airlangga University, Surabaya, East Java, Indonesia.

The rabbits were kept in cages according to the Guide for the Care and Use of Laboratory Animals [19] and were housed individually (size 100×60×75 cm) under a 12 h light-dark cycle at a temperature of 21±2°C and 50-55% humidity. All of the rabbits received scheduled feed and drinking water *ad libitum*.

### Preparation of PRF

Blood was drawn and immediately put into a 10 ml sterile glass tube without anticoagulants (Vaculab, ONEMED, Sidoarjo, Indonesia). It was centrifuged at 2700 rpm for 12 min [13] using a Kubota Compact Model 2420 (Tokyo, Japan). The final form consisted of three layers: Serum (top), red blood cells (bottom), and PRF gel (middle). The PRF was centrifuged at 2500 rpm for 5 min to collect the supernatant serum [20] for examination of the GF. The PRF was then cut into squares measuring 1×1 cm, at which time, it was ready to be seeded with SMSCs.

### Isolation and characterization of SMSCs

A combination of ketamine 50 mg/kg (Ket-A 100, Netherlands) and xylazine 2% 10 mg/kg IM (Xyla, Netherlands) was used for the rabbit anesthesia. The rabbit’s dorsal hair was removed using a razor, and its skin was disinfected using 70% alcohol and iodopovidone. The skin was then excised and washed with phosphate buffer saline (PBS) (Gibco, USA). Next, it was minced and added to collagenase type IV 0.25% (Worthington, USA). It was then incubated in 5% CO_2_ at 37°C for 45 min. Fetal bovine serum (FBS) 10% was added to the skin as a stopper and subsequently filtered and washed twice with PBS. The supernatant was then discarded, and a medium culture containing MEM alpha (Gibco, USA), 10% FBS (Gibco, USA), and 1% penicillin/streptomycin (Gibco, USA) was added. The cell suspension was cultured in a 60 mm dish and incubated in 5% CO_2_ at 37°C. This medium culture was changed every 3 days until 60-80% confluent and was subsequently subjected to passage for 4 times.

Characterization was performed using immunohistochemistry to immunophenotype the MSC surface markers. First, the cells were detached, separated into single cells, and placed with 20 μl of MSCs on a coverslip. Fixation was performed with 3% formaldehyde, and blocking was done with 1% FBS. Monoclonal antibody (BIOS, US) cluster of differentiation (CD) 73, CD90, CD105, and CD45 that had been labeled fluorescein isothiocyanate (Sigma-Aldrich, US) were added to each sample. They were then incubated at 37°C for 45 min. Finally, results were observed with a fluorescence microscope (Olympus IX71, Japan).

### Seeding PRF with SMSCs

The fourth passage cells were detached from the 60 mm culture dish using TrypLE Express 0.25% (Gibco). Cell counting was performed using a TC20 automated cell counter (California, USA). The PRF was seeded with as much as 5×10^4^ cells and incubated in 5% CO_2_ at 37°C. The PRF was collected on days 1, 3, and 5 after being seeded with SMSCs for scanning electron microscopy (SEM) examination. The supernatant medium culture at day 5 was collected for GF examination.

### Examination of GF by enzyme-linked immunosorbent assay (ELISA)

The supernatant serum PRF (Group A) and supernatant medium culture SMSCs on PRF (Group D) were collected to examine the levels of PDGF, IGF, VEGF, and TGF-β using an ELISA sandwich kit (Bioassay Technology Laboratory, China). Optical density was measured using a GloMAX Explorer multimode microplate reader (Promega, Wisconsin, USA).

### SEM examination

Examination was carried out by comparing the four groups with different seeding times. Group A consisted of PRF alone, Group B had PRF+SMSCs that were incubated for 1 day, Group C had PRF+SMSCs that were incubated for 3 days, and Group D had PRF+SMSCs that were incubated for 5 days.

To prepare samples for SEM analysis, the PRF was immersed in 4% paraformaldehyde in 0.1 M of PBS (pH 7.4) for 2 h at room temperature. Next, the samples were washed again with PBS and incubated with 8% formaldehyde at 4°C. After 2 days, the samples were fixed in osmium tetroxide and washed in PBS. The samples were dehydrated in graded alcohol of 30%, 50%, 70%, 80%, 90%, and 100% (twice), with each dehydration lasting for 10 min. Each sample was incubated in Bis(trimethylsilyl)amine (also known as hexamethyldisilazane and HMDS) for 10 min, airdried in a desiccator, and mounted on a suitably sized plastic microscope slide [21]. Some samples were randomly selected to be coated with gold using a sputter coating machine. SEM (ZEISS EVO^®^ MA 10, Germany) examination with 500× was then carried out. SEM analysis was conducted in the Laboratory for Energy and the Environment, Institut Teknologi Sepuluh Nopember, Surabaya.

### Statistical analysis

All the samples were tested for normality using the Shapiro–Wilk test, and p>0.05 was considered normal distribution. Statistical analysis was then performed to compare the mean values through unpaired t-test using Graph Prism 8.0.1 (San Diego, California, USA) software, with p<0.01 considered to be significant. The data are presented as mean±standard deviation (SD).

## Results

### Isolation and characterization of SMSCs

**Figure 1:**
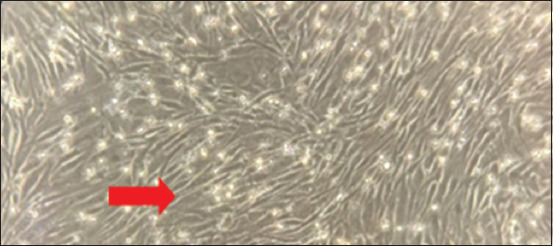
Morphology of the skin-derived mesenchymal stem cells at 100×; fibroblast-like cells, spindle-shaped cells (red arrow), and 90% confluence at the fourth passage.

**Figure 2:**
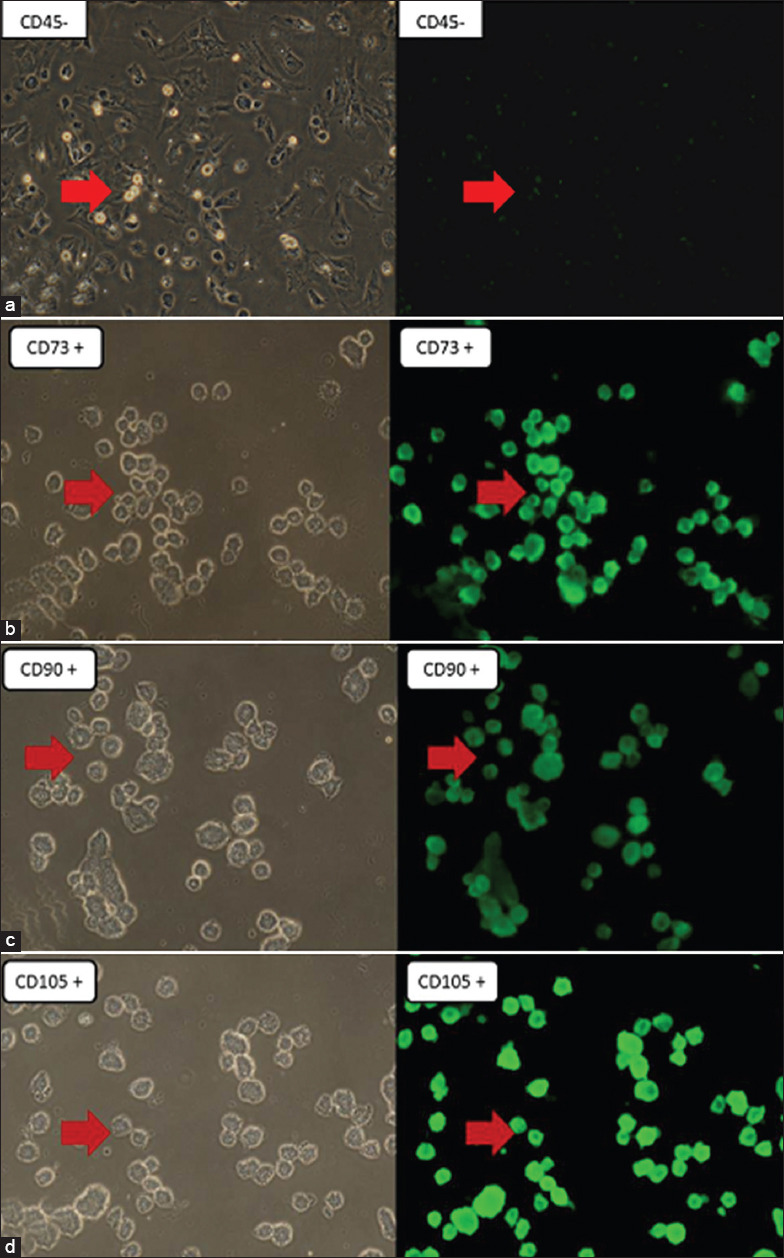
Microscopy of single skin-derived mesenchymal stem cells with fluorescein isothiocyanate-labeled antibody (a). CD45-, (b). CD73+, (c). CD90+, (d). CD105+ (red arrow). Left: Under inverted microscope. Right: Under fluorescent microscope (100×).

SMSCs demonstrated very high proliferation and rapid growth and reached 90% confluence. The culture of SMSCs had morphologically fibroblast-like cells, basically short or long and spindle shaped, forming small colonies with several triangular and polygonal cells (Figure-1). Cells at the fourth passage showed negative results against the CD45 marker and positive results against the CD73, CD90, and CD105 markers (Figure-2).

### SEM examination

**Figure 3:**
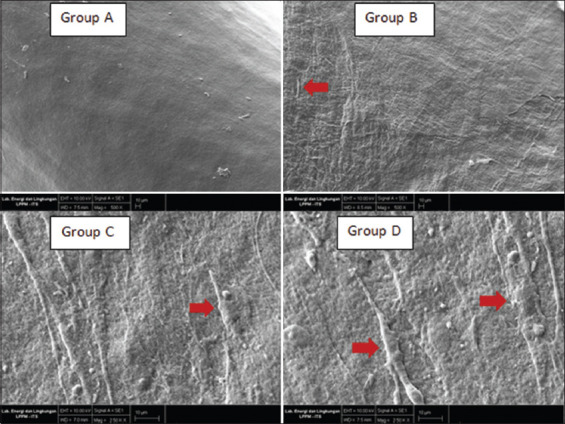
Results of scanning electron microscopy photography at 500× (Groups A and B) and 2500× (Groups C and D). Group A: Smooth surface and no cell formation. Groups B and C: Rough surface and a sparse skin-derived mesenchymal stem cells (SMSCs) formation. Group D: SMSCs covered the platelet-rich fibrin surface and showed elongated and pseudopodic cells (red arrow).

SEM examination at 500×, as shown in Figure-3, revealed that the surface of Group A (PRF alone) did not have any cell formation. However, there were some masses of platelet-like structures, and the surface appeared smooth and dense. For Group B (PRF+SMSCs and 1 day incubation) and Group C (PRF+SMSCs and 3 days of incubation), the surface appeared rougher, and the SMSCs could be seen on the surface of the PRF in an amount that was less than that of Group D (PRF+SMSCs and 5 days of incubation), in which the morphologies of the SMSCs were visibly elongated, and many cells’ pseudopodia showed good biocompatibility and attachment to the PRF. Group D PRF was covered with SMSCs that had been fused along with the PRF. The distribution of SMSCs in Group D was more organized than in the other groups. Moreover, all the groups characterized PRF with a highly condensed fibrin fiber network.

### Growth factor level analysis

On subjecting the data to the Shapiro–Wilk test, p>0.05 (Table-1) was found for each group, indicating that all samples were normally distributed.

**Table 1:** A Shapiro–Wilk test revealed that p value of each group was higher than 0.05, which indicated a normal distribution (n=8).

Group	p-value			

	PDGF	IGF	VEGF	TGF-β
Group A (PRF alone)	0.819	0.899	0.939	0.825
Group D (PRF+SMSCs 5 days incubation)	0.791	0.856	0.931	0.93
* Normal distribution	*	*	*	*

* Normal distribution at p>0.05. PDGF=Platelet-derived growth factor, IGF=Insulin-like growth factor, VEGF=Vascular endothelial growth factor, TGF-β=Transforming growth factor-β, PRF=Platelet-rich fibrin, SMSCs=Skin-derived mesenchymal stem cells

The levels of PDGF, IGF, VEGF, and TGF-β in Groups A (PRF alone) and D (PRF+SMSCs and 5 days of incubation) are shown in Table-2 with mean±SD for each GF. The levels of every GF were significantly higher in Group D (PRF+SMSCs and 5 days of incubation) than in Group A (PRF alone) (p<0.01).

**Table 2:** Description of Group A and Group D mean±SD results of an unpaired t-test between two groups (n=8).

Group	Mean±Standard deviation			

	PDGF (ng/ml)	IGF (ng/ml)	VEGF (ng/L)	TGF-b (ng/L)
Group A (PRF alone)	3.11±0.55	10.94±1.44	833.45±92.5	515.63±90.66
Group D (PRF+SMSCs 5 days incubation)	5.72±1.14	13.36±1.18	1794.9±78.46	741.5±51.9
*Sig	0.0001	0.0025	0.0001	0.0001

* Significant at p<0.01. PDGF=Platelet-derived growth factor, IGF=Insulin-like growth factor, VEGF=Vascular endothelial growth factor, TGF-β=Transforming growth factor-β, PRF=Platelet-rich fibrin, SMSCs=Skin-derived mesenchymal stem cells

## Discussion

The isolation and characterization of MSCs from rabbit skin tissue showed fluorescent luminescence of the positive markers CD105, CD90, and CD73, while no fluorescence luminescence of the negative marker CD45. In accordance with the previous studies, those MSC markers were MSCs from various sources, such as bone marrow, adipose tissue, dermis, muscle, and umbilical cord blood, and from different species. The cultured SMSCs were similar to fibroblastic and spindle-shaped cells and showed rapid cell growth [15,17,22-24].

SEM analysis showed that the SMSCs had spread to cover the PRFs surface by the 5^th^ day of incubation, indicating that they were attached and spread evenly. The other groups showed SMSC attachment, but not as much as after the 5^th^ day. This shows that the SMSCs were able to adhere and proliferate on PRF. Moreover, our SEM analysis highlighted different SMSC distributions that became more organized by the end of the evaluation (day 5). The PRF showed highly condensed fibrin fiber networks, which indicated ideal physical and biological scaffold characteristics [25].

The morphology of the platelets was totally modified by aggregation and clotting processes. Therefore, a large aggregate of platelet fibrin polymers was identified [26]. SEM examination of human PRF demonstrated a homogeneous distribution of fibrin with interconnected fibrin fibers trapping different cells [27]. The PRF membrane is a third-generation membrane containing the GF of the cells trapped in its fibrin matrix [26]. This could create an environment that would be appropriate for the relatively protracted survival of adipose MSCs due to the PRFs abundance of GF that enhance cell adhesion, proliferation, and spread [28-31]. Furthermore, PRF has the ability to stimulate the proliferation of human periodontal ligament cells and dental pulp stem cells [32-34].

Self-regulation of the fibrin network slowly formed a high fibrillar aggregation in PRF. This resulted in the entrapment of GF to the binding domains of the fibrin molecules [35] and slowly released GF (up to approximately ≥7 days after isolation). The GF released by the granules included polypeptide cytokines, such as PDGF, VEGF, TGF, fibroblast GF, epidermal GF, hepatocyte GF, and IGF [36].

Growth factors such as PDGF, IGF, VEGF, and TGF-β can be found in PRF, and they can be improved with the addition of SMSCs. This is done by secreting GF into the supernatant culture medium, adding to the GF content already present in the PRF. SMSCs are known to secrete proliferative cytokines, such as VEGF, IGF, PDGF, and TGF-β, which promote cell growth [16,37,38].

Previous reports have confirmed the expression of the PDGF receptor in mesenchymal cells, which regulate changes in the MSCs phenotype to prevent differentiating through the pathway through downregulation of miR-145/miR-143, which puts and keeps these cells in the proliferative phase or migration [39]. MSCs are capable of expressing and secreting IGF, which maintains the basic activity of signaling ERK1/2, which is needed to improve self-renewal properties [40]. Furthermore, fetal SMSCs also produce VEGF, which remains stable both *in vitro* and *in vivo* [38,41]. TGF-β is secreted by MSCs that are involved in immunomodulation and maintains the signaling of cellular regulation activities such as apoptosis, inflammation, fibrosis, adipocyte differentiation, and pro-migration [42,43]. It has multiple functional roles in normal physiology, from the 1^st^ day of embryonic development to homeostasis in adult tissue. Activation or deletion as a genetic mutation could lead to the development of diseases such as musculoskeletal disorders, cancer, and fibrosis [44].

We have proven that PRF and SMSCs have abundant GF. Combining them *in vitro* increased GF levels, making them an alternative solution to the problems currently faced by veterinary regenerative medicine. Therefore, further *in vivo* experimentation is needed to study the immunological and regeneration mechanisms in animals.

## Conclusion

SMSCs are able to adhere to and spread evenly over PRF at 5 days of incubation. PRF contains abundant levels of PDGF, IGF, VEGF, and TGF-β, which can be further improved by seeding with SMSCs.

PRF and SMSCs contain abundant GF that play important roles in regeneration, so more *in vivo* experiments, such as in rabbit models, need to be conducted to understand the pathways and mechanisms of skin tissue inflammation and regeneration. Furthermore, PRF could be used as an alternative biological scaffold for tissue engineering in veterinary medicine.

## Authors’ Contributions

This study was designed, drafted, and a part of the Master Thesis of the first author under the guidance of FAR and SAS. ISI, DK, NE, and AD carried out the experiment. FAR, APN, and PP revised the manuscript. All authors have read and approved the final version of the manuscript.
